# Supplementary material for: Whole-proteome structure/function prediction in uropathogenic E. coli reveals previously missed host-microbe and microbe-phage interaction pathways
Source: PLoS Genet. 2026 Nov 3;22(11):e1012187. doi: 10.1371/journal.pgen.1012187 (PMC13750657; doi:10.1371/journal.pgen.1012187)
Supplement: Supplementary file 1 [file pgen.1012187.s001.docx]

**Text S1**: Details of the construction of **Dataset I** and **Dataset II**.

The construction process of **Dataset I** involved three steps: (1) collecting 9,977 proteins that received new GO annotations in the UniProt-GOA 2025-05 release but had no annotations in the same GO aspect in the 2024-04 release; (2) removing redundancy using CD-HIT at a 40% sequence identity threshold and 80% alignment coverage; and (3) fifty proteins were randomly selected from each group of targets determined by AlphaFold2, Modeller, LOMETS, D-I-TASSER, and DMFold according to the workflow, resulting in the final test set with 250 proteins.

The construction process of **Dataset II** involved: (1) based on the data processing and selection procedures for **Dataset I**, 500 proteins were selected, including an additional 250 proteins; (2) for each of these proteins, 1,000 structure models were generated by running AlphaFold2 200 times with different random seeds, 100 runs with default settings and 100 runs without MSA or template information; (3) those proteins for which the 1,000 models were distributed across the three pLDDT intervals: (0, 50], (50, 70], and (70, 100] were selected as test proteins. In total, 128 proteins met these criteria and were selected as the final **Dataset II** to investigate the relationship between pLDDT and the performance of StarFunc.
