# Supplementary material for: Whole-proteome structure/function prediction in uropathogenic E. coli reveals previously missed host-microbe and microbe-phage interaction pathways
Source: PLoS Genet. 2026 Nov 3;22(11):e1012187. doi: 10.1371/journal.pgen.1012187 (PMC13750657; doi:10.1371/journal.pgen.1012187)
Supplement: Supplementary file 2 [file pgen.1012187.s002.docx]

**Text S2**: Details of homology-based function transfer approach used in this study

For sequence-based method, query protein sequence is searched against the UniProt Gene Ontology Annotation (UniProt-GOA) database by BLASTp [1] to identify homologous templates with function annotations, and protein functions of templates are transferred to query protein based on the bit-score and sequence identify of BLASTp hit. The prediction confidence score (Cscore_seq_) for each GO term *q* is calculated as follows,

$$\text{Cscore}_{\text{seq}}\left( \text{q} \right)\text{=}\frac{\sum_{\text{k}\text{=1}}^{\text{K}\text{(}\text{q}\text{)}} \text{bitscore}_{\text{k}}\text{(}\text{q}\text{)}\text{×ID}_{\text{k}}\text{(}\text{q}\text{)}}{\sum_{\text{k}\text{=1}}^{\text{K}} \text{bitscore}_{\text{k}}\text{× ID}_{\text{k}}}$$

(S1)

where *bitscore_k_* and *ID_k_* are the bit-score and sequence identity of the *k*-th BLASTp hit, while *K* is the total number of hits with at least one GO term in the same GO aspect (Molecular Function, MF; Biological Process, BP; or Cellular Component, CC) as term *q*. Meanwhile, *bitscore_k_*(*q*), *ID_k_*(*q*) and *K*(*q*) are the corresponding values for templates with GO term *q*. Here, the sequence identity (*ID_k_*) for hit *k* is calculated as the equation S2:

$$\text{ID}_{\text{k}}\text{=}\frac{\text{Liden}_{\text{k}}}{\text{max(}\text{L}\text{,}\text{L}_{\text{k}}\text{)}}$$

(S2)

Here, *L* and *L_k_* are the length of the query and the *k*-th template sequence respectively, while *Liden_k_* is the number of identical amino acids between the two proteins in the BLASTp alignment. Based on our recent benchmark study [2], combining both bit-score and sequence identity yields better performance than using either metric alone.

When generating the UniProt-GOA sequence database, we only use experimental GO annotations as defined by CAFA [3] (evidence codes: EXP, IDA, IPI, IMP, IGI, IEP, HTP, HDA, HMP, HGI, HEP, TAS, and IC), excluding computational prediction such as those with evidence code IEA. Negative annotations indicating lack of protein function (with qualifier “NOT” or evidence codes “ND”) are also excluded. Parent GO terms are automatically propagated from the leaf terms annotated by UniProtGOA. Similar to the practice in previous CAFA challenges [3], protein binding-only templates, i.e., template proteins whose only Molecular Function (MF) leaf term is GO:0005515 “protein binding”, are not used for MF term prediction, because “protein binding” is a highly generic function description.

## **References**

1.	Camacho C, Coulouris G, Avagyan V, Ma N, Papadopoulos J, Bealer K, et al. BLAST plus : architecture and applications. BMC Bioinformatics. 2009;10. doi: 10.1186/1471-2105-10-421.

2.	Zhang CX, Freddolino L. A large-scale assessment of sequence database search tools for homology-based protein function prediction. Briefings in Bioinformatics. 2024;25(4). doi: 10.1093/bib/bbae349.

3.	Zhou NH, Jiang YX, Bergquist TR, Lee AJ, Kacsoh BZ, Crocker AW, et al. The CAFA challenge reports improved protein function prediction and new functional annotations for hundreds of genes through experimental screens. Genome Biology. 2019;20(1):244. doi: 10.1186/s13059-019-1835-8.
