# Supplementary material for: Whole-proteome structure/function prediction in uropathogenic E. coli reveals previously missed host-microbe and microbe-phage interaction pathways
Source: PLoS Genet. 2026 Nov 3;22(11):e1012187. doi: 10.1371/journal.pgen.1012187 (PMC13750657; doi:10.1371/journal.pgen.1012187)
Supplement: Supplementary file 3 [file pgen.1012187.s003.docx]

**Text S3**: Definition of the Accretion-Weighted F-Measure (wF_max_)

The metric wF_max_ represents the maximum value of the protein-centric, information accretion-weighted F-measure across all prediction thresholds (*τ*). It is defined as:

$$\text{w}\text{F}_{\text{max}}\text{ =}\max_{\text{τ}\text{∈}\text{(0,1]}} \text{(}\frac{\text{2wpr(}\text{τ}\text{) × wrc(}\text{τ}\text{)}}{\text{wpr(τ) + wrc(}\text{τ}\text{)}}\text{)}$$

(S3)

$$\text{wpr}\text{(}\text{τ}\text{) = }\frac{\text{1}}{\text{m}\text{(}\text{τ}\text{)}}\sum_{\text{i}\text{=1}}^{\text{m}\text{(}\text{τ}\text{)}} \frac{\sum_{\text{q}} \text{IA}\text{(}\text{q}\text{)×1(}\text{q}\text{∈}\text{P}_{\text{i}}\text{(}\text{τ}\text{)}\text{∧}\text{T}_{\text{i}}\text{)}}{\sum_{\text{q}} \text{IA(}\text{q}\text{)×1(}\text{q}\text{∈}\text{P}_{\text{i}}\text{(}\text{τ}\text{))}}$$

(S4)

$$\text{wrc}\text{(}\text{τ}\text{) = }\frac{\text{1}}{\text{n}}\sum_{\text{i}\text{=1}}^{\text{n}_{\text{e}}} \frac{\sum_{\text{q}} \text{IA}\text{(}\text{q}\text{)×1(}\text{q}\text{∈}\text{P}_{\text{i}}\text{(}\text{τ}\text{)}\text{∧}\text{T}_{\text{i}}\text{)}}{\sum_{\text{q}} \text{IA(}\text{q}\text{)×1(}\text{q}\text{∈}\text{T}_{\text{i}}\text{)}}$$

(S5)

$$\text{IA(}\text{q}\text{)=} \text{log}_{\text{2}}\text{(}\frac{\text{1 + }\left| \text{proteins with parent term(s) of }\text{q} \right|}{\text{1 + }\left| \text{proteins with term }\text{q} \right|}\text{)}$$

(S6)

where wpr(*τ*) and wrc(*τ*) denote the information accretion-weighted precision and recall, respectively, at a given prediction score threshold *τ*. *P_i_*(*τ*) represents the set of predicted GO terms for protein *i* with confidence scores equal to or greater than *τ*, and *T_i_* denotes the set of ground-truth GO terms for protein *i*. The value *m*(*τ*) indicates the number of proteins with at least one predicted GO term scoring above *τ*, while *n* is the total number of proteins in the benchmark. $\text{1}$ is the indicator function. *q* denotes the *q*-th GO term, and IA(*q*) refers to its information accretion [1].

## **References**

1.	Clark WT, Radivojac P. Information-theoretic evaluation of predicted ontological annotations. Bioinformatics. 2013;29(13):53-61. doi: 10.1093/bioinformatics/btt228.
