# Supplementary material for: Whole-proteome structure/function prediction in uropathogenic E. coli reveals previously missed host-microbe and microbe-phage interaction pathways
Source: PLoS Genet. 2026 Nov 3;22(11):e1012187. doi: 10.1371/journal.pgen.1012187 (PMC13750657; doi:10.1371/journal.pgen.1012187)
Supplement: Supplementary file 4 [file pgen.1012187.s004.docx]

**Table S1.** The details of 17 common GO terms that were excluded due to their high prevalence.

| Aspects of GO terms | GO term numbers | Description |
| --- | --- | --- |
| Biological process | GO:0008150 | Biological process |
|  | GO:0009987 | Cellular process |
|  | GO:0008152 | Metabolic process |
|  | GO:0044237 | Cellular metabolic process |
|  | GO:0044238 | Primary metabolic process |
|  | GO:0050896 | Response to stimulus |
| Molecular function | GO:0003674 | Molecular function |
|  | GO:0005488 | Binding |
|  | GO:0005515 | Protein binding |
|  | GO:0003824 | Catalytic activity |
|  | GO:0036094 | Small molecule binding |
|  | GO:0097159 | Organic cyclic compound binding |
|  | GO:1901363 | Heterocyclic compound binding |
|  | GO:0043167 | Ion binding |
| Cellular component | GO:0005575 | Cellular component |
|  | GO:0110165 | Cellular anatomical entity |
|  | GO:0005622 | Intracellular anatomical structure |
