# Supplementary material for: Whole-proteome structure/function prediction in uropathogenic E. coli reveals previously missed host-microbe and microbe-phage interaction pathways
Source: PLoS Genet. 2026 Nov 3;22(11):e1012187. doi: 10.1371/journal.pgen.1012187 (PMC13750657; doi:10.1371/journal.pgen.1012187)
Supplement: Supplementary file 5 [file pgen.1012187.s005.docx]

**Table S2.** The details of the obviously incorrect (for *E. coli*) GO terms excluded from further analysis.

| Aspects of GO terms | GO term numbers | Description |
| --- | --- | --- |
| Molecular function | GO:0043886 | Structural constituent of carboxysome shell |
| Cellular component | GO:0005739 | Mitochondrion |
|  | GO:0031981 | Nuclear lumen |
|  | GO:0005634 | Nucleus |
