# Supplementary material for: Whole-proteome structure/function prediction in uropathogenic E. coli reveals previously missed host-microbe and microbe-phage interaction pathways
Source: PLoS Genet. 2026 Nov 3;22(11):e1012187. doi: 10.1371/journal.pgen.1012187 (PMC13750657; doi:10.1371/journal.pgen.1012187)
Supplement: Supplementary file 6 [file pgen.1012187.s006.docx]

**Table S3.** Primer and test sequences used to construct UTI89 mutants.

| **name** | **position** | **sequence** |
| --- | --- | --- |
| fur | left | 5’-TCAGGCTGGCTTATTTGCCTTCGTGCGCGTGCTCAT  CTTCGCGGCAATCGGTGTAGGCTGGAGCTGCTTC-3’ |
|  | right | 5’-AGTAACAGGACAGATTCCGCATGACTGATAACAAT  ACCGCCCTAAAGAAACATATGAATATCCTCCTTAG-3’ |
| fur test | left | 5’-AGGCAACGCAAACCGGAAAT-3’ |
|  | right | 5’-CAAGTGGCCTTGCCGTTGTA-3’ |
| ybtE | left | 5’-ATGAATTCTTCCTTTGAATCTCTGATTGAACAGTAT  CCCTTACCCATTGCGTGTAGGCTGGAGCTGCTTC-3’ |
|  | right | 5’-TATTTCAACCTGTTTCGGGTCGGTTTGCGCTTATT  GGGCAGAATGGCGATCATATGAATATCCTCCTTAG-3’ |
| ybtE test | left | 5’-ACGCAGATTGTCCGCCATTT-3’ |
|  | right | 5’-CCCTGTAAGACGGCGAAACG-3’ |
| fyuA | left | 5’-TCTTACAGGGACTCACAACAATGAAAATGACACGG  CTTTATCCTCTGGCCCATATGAATATCCTCCTTAG-3’ |
|  | right | 5’-tcagaagaaatcaattcgcgtattgataccgacggt  gcgacccatattgagtgtaggctggagctgcttc-3’ |
| fyuA test | left | 5’-ACCGACCCGAAACAGGTTGA-3’ |
|  | right | 5’-CCGAATGATTTTCGGGGTGA-3’ |
