# Supplementary material for: Whole-proteome structure/function prediction in uropathogenic E. coli reveals previously missed host-microbe and microbe-phage interaction pathways
Source: PLoS Genet. 2026 Nov 3;22(11):e1012187. doi: 10.1371/journal.pgen.1012187 (PMC13750657; doi:10.1371/journal.pgen.1012187)
Supplement: Supplementary file 7 [file pgen.1012187.s007.docx]

**Table S4.** Bacterial strains used in this study.

| Strain | Description | Reference |
| --- | --- | --- |
| UTI89 | human cystitis isolate | ref Mulvey 2001 [1] |
| UTI89Δ*fur* | *fur* knockout | this study |
| UTI89Δ*entB* | *entB* knockout | ref Henderson 2009 [2] |
| UTI89Δ*iroB* | *entB* knockout | ref Henderson 2009 [2] |
| UTI89Δ*ybtS* | *ybtS* knockout | ref Henderson 2009 [2] |
| UTI89Δ*ybtE* | *ybtE* knockout | this study |
| UTI89Δ*yfuA* | *fyuA* knockout | this study |

## **References**

1.	Mulvey MA, Schilling JD, Hultgren SJ. Establishment of a persistent *Escherichia coli* reservoir during the acute phase of a bladder infection. Infection and Immunity. 2001;69(7):4572-9. doi: 10.1128/IAI.69.7.4572-4579.2001. PubMed PMID: WOS:000169335700048.

2.	Henderson JP, Crowley JR, Pinkner JS, Walker JN, Tsukayama P, Stamm WE, et al. Quantitative Metabolomics Reveals an Epigenetic Blueprint for Iron Acquisition in Uropathogenic *Escherichia coli*. PLOS Pathogens. 2009;5(2). doi: 10.1371/journal.ppat.1000305. PubMed PMID: WOS:000263928000016.
