# Supplementary material for: Whole-proteome structure/function prediction in uropathogenic E. coli reveals previously missed host-microbe and microbe-phage interaction pathways
Source: PLoS Genet. 2026 Nov 3;22(11):e1012187. doi: 10.1371/journal.pgen.1012187 (PMC13750657; doi:10.1371/journal.pgen.1012187)
Supplement: Supplementary file 8 [file pgen.1012187.s009.docx]

**Table S6.** Functional annotation rates of whole UTI89 genome across annotation score categories for biological process.

|  | Score 1 |  | Score 2 |  | Score 3 |  | Score 4 |  | Score 5 |
| --- | --- | --- | --- | --- | --- | --- | --- | --- | --- |
| StarFunc only | 39.3% |  | 25.2% |  | 10.3% |  | 6.2% |  | 1.8% |
| Common | 14.7% |  | 50.8% |  | 81.4% |  | 90.4% |  | 96.5% |
| UniProt only | 3.5% |  | 7.5% |  | 4.6% |  | 1.8% |  | 1.0% |
| Unannotated | 42.5% |  | 16.5% |  | 3.7% |  | 1.6% |  | 0.7% |
