# Supplementary material for: Whole-proteome structure/function prediction in uropathogenic E. coli reveals previously missed host-microbe and microbe-phage interaction pathways
Source: PLoS Genet. 2026 Nov 3;22(11):e1012187. doi: 10.1371/journal.pgen.1012187 (PMC13750657; doi:10.1371/journal.pgen.1012187)
Supplement: Supplementary file 9 [file pgen.1012187.s010.docx]

**Table S7.** Functional annotation rates of whole UTI89 genome across annotation score categories for cellular component.

|  | Score 1 |  | Score 2 |  | Score 3 |  | Score 4 |  | Score 5 |
| --- | --- | --- | --- | --- | --- | --- | --- | --- | --- |
| StarFunc only | 75.2% |  | 33.1% |  | 24.6% |  | 15.0% |  | 9.2% |
| Common | 22.7% |  | 66.6% |  | 75.4% |  | 85.0% |  | 90.8% |
| UniProt only | 0.2% |  | - |  | - |  | - |  | - |
| Unannotated | 1.9% |  | 0.3% |  | - |  | - |  | - |
