# Supplementary material for: Whole-proteome structure/function prediction in uropathogenic E. coli reveals previously missed host-microbe and microbe-phage interaction pathways
Source: PLoS Genet. 2026 Nov 3;22(11):e1012187. doi: 10.1371/journal.pgen.1012187 (PMC13750657; doi:10.1371/journal.pgen.1012187)
Supplement: Supplementary file 10 [file pgen.1012187.s011.docx]

**Table S8.** Functional annotation rates of whole UTI89 genome across annotation score categories for molecular function.

|  | Score 1 |  | Score 2 |  | Score 3 |  | Score 4 |  | Score 5 |
| --- | --- | --- | --- | --- | --- | --- | --- | --- | --- |
| StarFunc only | 20.4% |  | 17.1% |  | 7.1% |  | 2.8% |  | 0.3% |
| Common | 21.3% |  | 56.2% |  | 74.7% |  | 88.9% |  | 96.5% |
| UniProt only | 3.9% |  | 6.0% |  | 6.7% |  | 3.9% |  | 1.7% |
| Unannotated | 54.4% |  | 20.7% |  | 11.5% |  | 4.4% |  | 1.5% |
