# Supplementary material for: Whole-proteome structure/function prediction in uropathogenic E. coli reveals previously missed host-microbe and microbe-phage interaction pathways
Source: PLoS Genet. 2026 Nov 3;22(11):e1012187. doi: 10.1371/journal.pgen.1012187 (PMC13750657; doi:10.1371/journal.pgen.1012187)
Supplement: Supplementary file 14 [file pgen.1012187.s015.docx]

**Table S12.** Functional annotation rates of UTI89 proteins excluding phage-derived proteins across annotation score categories for biological process.

|  | Score 1 |  | Score 2 |  | Score 3 |  | Score 4 |  | Score 5 |
| --- | --- | --- | --- | --- | --- | --- | --- | --- | --- |
| StarFunc only | 38.8% |  | 25.7% |  | 10.4% |  | 6.1% |  | 1.8% |
| Common | 14.2% |  | 50.4% |  | 81.3% |  | 90.5% |  | 96.5% |
| UniProt only | 3.6% |  | 7.0% |  | 4.6% |  | 1.8% |  | 0.9% |
| Unannotated | 43.4% |  | 16.9% |  | 3.7% |  | 1.6% |  | 0.8% |
