# Supplementary material for: Whole-proteome structure/function prediction in uropathogenic E. coli reveals previously missed host-microbe and microbe-phage interaction pathways
Source: PLoS Genet. 2026 Nov 3;22(11):e1012187. doi: 10.1371/journal.pgen.1012187 (PMC13750657; doi:10.1371/journal.pgen.1012187)
Supplement: Supplementary file 19 [file pgen.1012187.s020.docx]

**Table S17.** Functional annotation rates of UTI89 phage-derived proteins across annotation score categories for molecular function.

|  | Score 1 |  | Score 2 |  | Score 3 |  | Score 4 |  | Score 5 |
| --- | --- | --- | --- | --- | --- | --- | --- | --- | --- |
| StarFunc only | 27.1% |  | - |  | - |  | - |  | - |
| Common | 19.9% |  | 87.0% |  | 100.0% |  | 100.0% |  | 100.0% |
| UniProt only | 6.4% |  | 8.7% |  | - |  | - |  | - |
| Unannotated | 46.6% |  | 4.3% |  | - |  | - |  | - |
