# Supplementary material for: Whole-proteome structure/function prediction in uropathogenic E. coli reveals previously missed host-microbe and microbe-phage interaction pathways
Source: PLoS Genet. 2026 Nov 3;22(11):e1012187. doi: 10.1371/journal.pgen.1012187 (PMC13750657; doi:10.1371/journal.pgen.1012187)
Supplement: Supplementary file 20 [file pgen.1012187.s021.docx]

**Table S18.** pLDDT scores of the 5 proteins predicted by DMFold that also have available structure models in the AlphaFold Database (AFDB).

| UinProt ID |  | pLDDT |  |
| --- | --- | --- | --- |
|  | DMFold |  | AFDB |
| Q1RD73 | 58.93 |  | 62.31 |
| Q1R2T5 | 70.46 |  | 68.96 |
| Q1R1Q5 | 74.17 |  | 69.28 |
| Q1R286 | 73.86 |  | 50.22 |
| Q1RE11 | 67.98 |  | 56.90 |
| Average | 69.08 |  | 61.53 |
| Median | 70.46 |  | 62.31 |
