# Supplementary material for: Whole-proteome structure/function prediction in uropathogenic E. coli reveals previously missed host-microbe and microbe-phage interaction pathways
Source: PLoS Genet. 2026 Nov 3;22(11):e1012187. doi: 10.1371/journal.pgen.1012187 (PMC13750657; doi:10.1371/journal.pgen.1012187)
Supplement: Supplementary file 21 [file pgen.1012187.s022.docx]

**Table S19.** pLDDT scores of the 13 proteins predicted by DMFold that have no available structure models in the AFDB.

| UinProt ID |  | pLDDT |  |
| --- | --- | --- | --- |
|  | DMFold |  | AFDB |
| Q1RAC4 | 79.19 |  | - |
| Q1R2R5 | 85.09 |  | - |
| Q1RAD0 | 83.29 |  | - |
| Q1RAD2 | 80.73 |  | - |
| Q1RAD3 | 82.77 |  | - |
| Q1RAF9 | 83.70 |  | - |
| Q1RE24 | 65.83 |  | - |
| Q1R4Z3 | 87.44 |  | - |
| Q1R728 | 82.46 |  | - |
| Q1R8M6 | 91.82 |  | - |
| Q1RAD6 | 83.51 |  | - |
| Q1RC09 | 88.05 |  | - |
| Q1RFP2 | 82.43 |  | - |
| Average | 82.79 |  | - |
| Median | 83.29 |  | - |
