# Supplementary material for: Whole-proteome structure/function prediction in uropathogenic E. coli reveals previously missed host-microbe and microbe-phage interaction pathways
Source: PLoS Genet. 2026 Nov 3;22(11):e1012187. doi: 10.1371/journal.pgen.1012187 (PMC13750657; doi:10.1371/journal.pgen.1012187)
Supplement: Supplementary file 24 [file pgen.1012187.s026.docx]

**Table S23.** Functional annotation rates of UTI89 biofilm formation-related proteins across annotation score categories for molecular function.

|  | Score 1 |  | Score 2 |  | Score 3 |  | Score 4 |  | Score 5 |
| --- | --- | --- | --- | --- | --- | --- | --- | --- | --- |
| StarFunc only | 16.1% |  | 20.0% |  | 10.5% |  | - |  | - |
| Common | 35.5% |  | 40.0% |  | 63.2% |  | 75.0% |  | 93.9% |
| UniProt only | 6.5% |  | 20.0% |  | 5.3% |  | 12.5% |  | 6.1% |
| Unannotated | 41.9% |  | 20.0% |  | 21.0% |  | 12.5% |  | - |
