# Supplementary material for: Whole-proteome structure/function prediction in uropathogenic E. coli reveals previously missed host-microbe and microbe-phage interaction pathways
Source: PLoS Genet. 2026 Nov 3;22(11):e1012187. doi: 10.1371/journal.pgen.1012187 (PMC13750657; doi:10.1371/journal.pgen.1012187)
Supplement: Supplementary file 27 [file pgen.1012187.s029.docx]

**Table S26.** Functional annotation rates of UTI89 proteins differentially expressed in biofilm-like intracellular bacterial communities (IBCs) across annotation score categories for molecular function.

|  | Score 1 |  | Score 2 |  | Score 3 |  | Score 4 |  | Score 5 |
| --- | --- | --- | --- | --- | --- | --- | --- | --- | --- |
| StarFunc only | 25.9% |  | - |  | 12.5% |  | - |  | - |
| Common | 7.4% |  | 75.0% |  | 62.5% |  | 100.0% |  | 100.0% |
| UniProt only | 3.7% |  | - |  | - |  | - |  | - |
| Unannotated | 63.0% |  | 25.0% |  | 25.0% |  | - |  | - |
