# Supplementary material for: Whole-proteome structure/function prediction in uropathogenic E. coli reveals previously missed host-microbe and microbe-phage interaction pathways
Source: PLoS Genet. 2026 Nov 3;22(11):e1012187. doi: 10.1371/journal.pgen.1012187 (PMC13750657; doi:10.1371/journal.pgen.1012187)
Supplement: Supplementary file 28 [file pgen.1012187.s041.docx]

**Response to Reviewer #1’s comments**

We very much appreciate the insightful comments from the Reviewers, which were very helpful in improving the quality and presentation of the manuscript. The Reviewers’ primary concerns focused on strengthening the methodological validation of StarFunc, including its prediction reliability, benchmarking against recent state-of-the-art methods, computational efficiency, and reproducibility. Below, we provide point-by-point responses to each of the Reviewers’ comments. All corresponding changes have been highlighted in yellow in the revised manuscript.

1. **The Reviewer commented:**

***Figure 2 B, StarFunc alone predicts between ~23 and 44 % of all Go-Terms. It would be useful for readers to assess the reliability of StarFunc predictions at scale if the authors showed a Cscore distribution for the GO-Terms predicted by StarFunc alone, and those present in the UniProt annotation and predicted by StarFunc (both).***

We thank the reviewer for this valuable suggestion. To assess the confidence of StarFunc predictions at the proteome scale, we compared the StarFunc CScore distributions of protein-GO term pairs classified as “StarFunc alone” with those classified as “both,” i.e., GO terms present in the UniProt annotation and also predicted by StarFunc. The distributions were analyzed separately for biological process (BP), cellular component (CC), and molecular function (MF).

**Fig. S1.** Distribution of StarFunc confidence scores for StarFunc-only and UniProt-supported predictions across GO aspects.

We have added aspect-specific kernel density plots showing the two distributions (**Fig. S1**), with the sample size and median CScore indicated for each group. We also provide the corresponding summary statistics as supplementary data.

For BP, the analysis included 11,584 StarFunc-alone and 31,670 “both” protein-GO term pairs. The median CScore was 0.916 for StarFunc alone and 0.961 for both, with interquartile ranges of 0.740-0.980 and 0.878-0.989, respectively. For CC, the median CScore was 0.946 for 14,796 StarFunc-alone pairs and 1.000 for 7,434 both pairs; the respective interquartile ranges were 0.764-0.999 and 0.989-1.000. For MF, the median CScore was 0.932 for 5,132 StarFunc-alone pairs and 1.000 for 20,558 both pairs, with interquartile ranges of 0.703-0.999 and 0.998-1.000.

In all three GO aspects, the CScore distribution of the both group was significantly shifted toward higher values compared with the StarFunc-alone group (all p < 0.001). These results show that StarFunc predictions supported by existing UniProt annotations generally receive higher confidence scores, particularly for CC and MF. Nevertheless, the median CScore of the StarFunc-alone group exceeded 0.9 in all three aspects. Thus, although these predictions are not currently represented in the UniProt annotation, a substantial proportion have high StarFunc confidence and may represent plausible functional annotations that warrant further experimental. We have incorporated these distribution plots and the associated quantitative results into the revised manuscript.

In comparison with the original function annotations provided by UniProt, 29,232 specific GO annotations were unique to UniProt, 38,753 were shared between UniProt and StarFunc, and 52,421 specific GO annotations were provided only by StarFunc (Figure 2B). Here, the GO terms provided by UniProt are from the UniProt-GOA project [43]. Even after updating the function annotations of UTI89 proteome using GO terms from highly similar and better-annotated proteins in the K12 strain, StarFunc still can provide 31,512 additional GO term annotations (Figure 2B). Among these GO annotations, 5,132 pertained to MF, 11,584 to BP, and 14,796 to CC (Figure 2C). Although GO terms supported by both UniProt and StarFunc generally had higher CScores, StarFunc-only predictions also showed high confidence, with median CScores above 0.9 across all three GO aspects (**Supplementary Figure S1**). We further examined the UniProt-only annotations to determine whether they were completely absent from the StarFunc predictions or instead ranked below the reporting threshold. Of the 43,381 UniProt-only protein-GO term pairs, 40,046 (92.31%) were recovered among the unthresholded StarFunc predictions, whereas only 3,335 (7.69%) were not recovered. The recovered terms had a median rank difference of 14 positions relative to the lowest-ranked reported GO term from the same GO aspect, indicating that most UniProt-only annotations were present in the StarFunc candidate lists but were excluded by the CScore ≥ 0.5 reporting threshold (**Supplementary Figure S2**).

2. **The Reviewer commented:**

***Similarly, Figure 2 B shows that StarFunc fails to predict UniProt Go annotations in between ~ 25 and 33% of cases. In cases where StarFunc failed to predict the correct UniProt annotation (UniProt), it would be useful to compare the Cscore of the UniProt annotations as predicted by StarFunc for a given protein to that of the reported annotations. Essentially a rank difference between the uniport annotation vs StarFunc annotation.***

We thank the reviewer for this helpful suggestion. In our original analysis, only StarFunc predictions with CScore ≥ 0.5 were retained. Therefore, a GO term classified as “UniProt-only” did not necessarily indicate that StarFunc had completely failed to recover the annotation; in many cases, the term could have been predicted with a CScore below the reporting threshold.

**Figure S2.** Heatmaps of UniProt-only GO terms according to UniProt annotation score and minimum rank difference from StarFunc-reported GO terms.

To address this issue, we repeated the analysis using all StarFunc predictions with CScore ≥ 0.0. For each protein-GO term pair previously classified as UniProt-only, we determined its StarFunc CScore and its rank among all StarFunc predictions from the same GO aspect (BP, CC, or MF). We then compared it with the cutoff-boundary GO term, defined as the lowest-scoring prediction with CScore ≥ 0.5 for the same protein and GO aspect. Rank differences and CScore differences were calculated relative to this boundary term.

Among 43,381 UniProt-only protein-GO term pairs, 3,335 pairs (7.69%), involving 965 proteins, were not recovered by StarFunc even at CScore ≥ 0.0. The remaining 40,046 pairs (92.31%) were recovered with nonzero but sub-threshold CScores. Relative to the CScore ≥ 0.5 reporting boundary, the median rank difference was 14 positions. The proportions of completely unrecovered terms were 9.93% for BP, 4.42% for CC, and 3.99% for MF. We have added three heatmaps (all in **Fig. S2**) showing the relationships between the UniProt-only term rank difference and the protein annotation score. When the three GO aspects were examined separately, distinct patterns were observed. For biological process (BP), 24,135 of 26,797 UniProt-only protein-GO term pairs (90.07%) were recovered with sub-threshold CScores, whereas 2,662 pairs (9.93%) were not recovered. The recovered BP terms had a median rank difference of 14 positions. BP therefore showed the highest proportion of completely unrecovered annotations and the broadest rank-difference distribution, indicating that some UniProt BP terms were placed substantially lower in the StarFunc candidate lists. For cellular component (CC), 2,640 of 2,762 pairs (95.58%) were recovered below the threshold, while 122 pairs (4.42%) were not recovered. The median rank difference was 9 positions. Thus, UniProt-only CC terms generally occurred closer to the reporting boundary in rank than BP or MF terms, although their confidence scores were more clearly separated from those of the reported CC predictions. For molecular function (MF), 13,271 of 13,822 pairs (96.01%) were recovered with sub-threshold scores, and 551 pairs (3.99%) were not recovered. MF had a median rank difference of 14 positions. These results indicate that StarFunc recovered the largest proportion of UniProt-only annotations for MF but generally assigned them low confidence. Overall, 40,046 of the 43,381 UniProt-only protein–GO term pairs (92.31%) were present among the unthresholded StarFunc predictions, whereas only 3,335 pairs (7.69%) were completely unrecovered. The recovered terms had a median rank difference of 14 positions relative to the lowest-ranked reported term from the same GO aspect. These findings demonstrate that most annotations originally classified as UniProt-only were not entirely absent from the StarFunc results. Instead, StarFunc recovered them as lower-confidence predictions that were excluded by the CScore ≥ 0.5 reporting threshold.

In comparison with the original function annotations provided by UniProt, 29,232 specific GO annotations were unique to UniProt, 38,753 were shared between UniProt and StarFunc, and 52,421 specific GO annotations were provided only by StarFunc (Figure 2B). Here, the GO terms provided by UniProt are from the UniProt-GOA project [43]. Even after updating the function annotations of UTI89 proteome using GO terms from highly similar and better-annotated proteins in the K12 strain, StarFunc still can provide 31,512 additional GO term annotations (Figure 2B). Among these GO annotations, 5,132 pertained to MF, 11,584 to BP, and 14,796 to CC (Figure 2C). Although GO terms supported by both UniProt and StarFunc generally had higher CScores, StarFunc-only predictions also showed high confidence, with median CScores above 0.9 across all three GO aspects (**Supplementary Figure S1**). We further examined the UniProt-only annotations to determine whether they were completely absent from the StarFunc predictions or instead ranked below the reporting threshold. Of the 43,381 UniProt-only protein-GO term pairs, 40,046 (92.31%) were recovered among the unthresholded StarFunc predictions, whereas only 3,335 (7.69%) were not recovered. The recovered terms had a median rank difference of 14 positions relative to the lowest-ranked reported GO term from the same GO aspect, indicating that most UniProt-only annotations were present in the StarFunc candidate lists but were excluded by the CScore ≥ 0.5 reporting threshold (**Supplementary Figure S2**).

3. **The Reviewer commented:**

***In Figure 3, the authors compare their StarFunc pipeline to two alternatives. The authors also claim to meet or exceed state of the art methods with StarFunc, however the authors fail to compare to more recent pipelines, such as NetGO 4.0 or GOAnnotator.***

Thank you for this important suggestion. We agree that comparison with more recent functional annotation methods strengthens the evaluation of StarFunc. We have therefore extended Figure 3 to include GOAnnotator and NetGO 3.0, using the same 250 target proteins, ground-truth annotations, information-accretion weights, and wFmax evaluation procedure applied to all other methods. GOAnnotator was evaluated using literature, protein metadata, and candidate GO annotations restricted to information available before the April 2024 benchmark cutoff. Across the molecular function (MF), biological process (BP), and cellular component (CC) aspects, GOAnnotator achieved wFmax values of 0.390, 0.292, and 0.483, respectively, with an average of 0.388. NetGO 3.0 achieved corresponding values of 0.438, 0.369, and 0.583, with an average of 0.464. StarFunc achieved an average wFmax of 0.535 and therefore outperformed GOAnnotator and NetGO 3.0 by 37.9% and 15.5%, respectively. The revised figure reports 95% confidence intervals calculated by 10,000-resample bootstrap analysis.

We also evaluated NetGO 4.0 for completeness. It achieved wFmax values of 0.640, 0.573, and 0.630 for MF, BP, and CC, respectively, corresponding to an average of 0.614. When anonymized sequence identifiers were used, thereby preventing use of the literature-based component associated with protein identifiers, the average wFmax increased to 0.734 (MF: 0.807, BP: 0.712, CC: 0.683). However, we did not include NetGO 4.0 as a directly comparable baseline in Figure 3 because our evaluation set was defined using annotations accumulated from April 16, 2024 onward, whereas NetGO 4.0 was released on February 10, 2025, and thus many of the proteins used in our test set are used in the NetGO 4.0 training set. In addition, NetGO 4.0 is currently available through a public server without publicly available code that would allow us to reproduce the method using databases frozen at our April 2024 benchmark cutoff. We therefore used NetGO 3.0 as the temporally controlled NetGO baseline for the primary comparison.

Overall, for the three GO aspects, our pipeline demonstrates superior performance in BP and CC compared to the better of the four baseline methods (Figure 3A), achieving a 2.3% higher wFmax for BP (0.445 vs. 0.435 for the sequence-based method; p=2.46×10-39, Wilcoxon signed rank test, which is also used throughout this work for similar comparisons unless otherwise noted) and an 8.7% improvement for CC (0.663 vs. 0.610 for DeepGOPlus; p=0.00). For MF, although the difference is statistically significant (p=1.24×10-5), both our pipeline and the sequence-based method achieve an identical average wFmax of 0.499, indicating comparable predictive performance in practice. Nevertheless, when averaging across the three GO aspects, our pipeline attains an average wF_max_ of 0.535, which is 4.7%, 5.9%, 15.4%, and 37.8% higher than that of the ‘sequence-based’ (0.511), DeepGOPlus (0.505), NetGO 3.0 (0.464), and GOAnnotator (0.388) pipelines, respectively. These results further highlight the overall robustness and effectiveness of our pipeline, which delivers consistently strong performance across GO aspects and achieves higher average functional annotation accuracy than homology-based, deep learning-based, and annotation-transfer methods. Runtime benchmarking under identical conditions (clean re-run from scratch on a single RTX 5090 GPU) showed average runtimes of 831.6, 94.7, 53.5, and 2.19 s per protein for StarFunc, the sequence-based method, DeepGOPlus, and GOAnnotator, respectively. Thus, there is a clear effort-accuracy tradeoff where StarFunc represents a relatively expensive, but higher accuracy, approach to annotation prediction – we note here, however, that the costs of StarFunc predictions are still quite small relative to the experimental effort likely to be invested for follow-up investigations of even a single target.

4. **The Reviewer commented:**

***In Figure 3, given the relatively modest improvements in wFmax observed for StarFunc, it would help the reader to also compare the computational load between methods tested. A metric such as sequences annotation per time, may be a useful metric for users who may have large sequence databases to predict.***

Thank you for this helpful suggestion. We agree that computational efficiency is an important practical consideration, particularly for large-scale protein annotation. We have therefore added the average runtime per protein and the corresponding annotation throughput for StarFunc, the sequence-based method, DeepGOPlus, and GOAnnotator.

For annotation of all three GO aspects, the complete StarFunc pipeline required an average of 831.6 s per protein, whereas the sequence-based method required 94.7 s per protein. DeepGOPlus required an average of 53.5 s per protein. GOAnnotator required an average of 2.19 s per protein. Overall, these results indicate that StarFunc achieves improved annotation accuracy at the expense of higher computational cost, reflecting its multiple complementary prediction modules and evidence-integration steps. In contrast, DeepGOPlus and GOAnnotator provide substantially higher annotation throughput and may be preferable when computational efficiency is the primary consideration.

Overall, for the three GO aspects, our pipeline demonstrates superior performance in BP and CC compared to the better of the four baseline methods (Figure 3A), achieving a 2.3% higher wFmax for BP (0.445 vs. 0.435 for the sequence-based method; p=2.46×10-39, Wilcoxon signed rank test, which is also used throughout this work for similar comparisons unless otherwise noted) and an 8.7% improvement for CC (0.663 vs. 0.610 for DeepGOPlus; p=0.00). For MF, although the difference is statistically significant (p=1.24×10-5), both our pipeline and the sequence-based method achieve an identical average wFmax of 0.499, indicating comparable predictive performance in practice. Nevertheless, when averaging across the three GO aspects, our pipeline attains an average wFmax of 0.535, which is 4.7%, 5.9%, 15.4%, and 37.8% higher than that of the ‘sequence-based’ (0.511), DeepGOPlus (0.505), NetGO 3.0 (0.464), and GOAnnotator (0.388) respectively. These results further highlight the overall robustness and effectiveness of our pipeline, which delivers consistently strong performance across GO aspects and achieves higher average functional annotation accuracy than homology-based, deep learning-based, and annotation-transfer methods. Runtime benchmarking under identical conditions (clean re-run from scratch on a single RTX 5090 GPU) showed average runtimes of 831.6, 94.7, 53.5, and 2.19 s per protein for StarFunc, the sequence-based method, DeepGOPlus, and GOAnnotator, respectively. Thus, there is a clear effort-accuracy tradeoff where StarFunc represents a relatively expensive, but higher accuracy, approach to annotation prediction – we note here, however, that the costs of StarFunc predictions are still quite small relative to the experimental effort likely to be invested for follow-up investigations of even a single target.

5. **The Reviewer commented:**

***In line 382, the authors mistakenly refer to Figure 1, where it should be Figure 2***

Thank you for pointing out this error. We have corrected it in the revised manuscript.

6. **The Reviewer commented:**

***The Authors should provide the date of access to the UniProt data used in this study.***

Thank you for pointing this out. We have added the UniProt data access date (July 2, 2024) in the revised manuscript.

**Data availability**

Protein sequences for the E. coli UTI89 proteome were collected from UniProt with proteome ID UP000001952. The GO annotations of these UniProt entries are collected from UniProt-GOA release 2024-07-02. All predicted structure models and function annotations are accessible through our public database at https://seq2fun.dcmb.med.umich.edu/UTI89.

**Response to Reviewer #2’s comments**

We very much appreciate the comments and suggestions from the Reviewer, which are helpful for improving the quality and presentation of the manuscript. The reviewer is primarily concerned with strengthening the biological interpretation and contextualization of the predicted functions, including comparisons with previous experimental studies, refinement of biological conclusions, clarification of terminology, and additional discussion of existing biological knowledge. In the text below, we include point-by-point replies to the comments of the Reviewer, where all changes have been highlighted in yellow in the manuscript.

1. **The Reviewer commented:**

***L80. Do you mean the ‘structure and function of bacterial ‘proteins’ essential for…***

Thank you for pointing this out. We agree that “proteins” is more appropriate than “genes” in this context. We have revised the sentence accordingly in the manuscript.

2. **The Reviewer commented:**

***L330. ‘only 4 proteins were assigned an annotation score of 5’. I found this hard to understand given we know so much about E. coli proteins. Further explanation would be helpful.***

Thank you for raising this important point. We agree that the low number of proteins with the highest UniProt annotation score (score 5) may appear surprising given that *E. coli* is one of the most extensively studied bacterial species. However, UniProt annotation scores are a heuristic that reflects the level of confidence, specificity, and completeness of available functional annotations rather than the overall biological knowledge of a species. A score of 5 represents the highest annotation level and is assigned only to proteins with highly curated and comprehensive functional information. Many well-studied proteins may still receive lower scores if their annotations are incomplete or lack sufficient experimental evidence.

In addition, although UTI89 and *E. coli* K-12 share high sequence similarity, UTI89 is a uropathogenic strain with strain-specific proteins, including many proteins encoded on virulence-associated mobile genetic elements, that remain poorly characterized. To further improve the annotation coverage, we transferred annotations from highly similar *E. coli* K-12 proteins (>90% sequence identity) as described in the manuscript. We have added further clarification of this point in the revised manuscript.

According to the raw data provided by UniProt, out of the 5,192 proteins in the UTI89 proteome, only 4 proteins were assigned an annotation score of 5 (<0.1%), 39 proteins were assigned a score of 4 (0.8%), 600 proteins were assigned a score of 3 (11.6%), 1,408 proteins had a score of 2 (27.1%), and 3,141 proteins were assigned a score of 1 (60.5%), as shown in the 3rd ring in **Figure 2A**. Although *E. coli* is a well-studied organism, UniProt annotation scores reflect the completeness, specificity, and confidence of individual protein functional annotations rather than the overall level of biological knowledge available for the species. Therefore, even proteins from extensively studied organisms may not receive the highest annotation score if their functional annotations remain incomplete or if there is a lack of sufficient experimental evidence for that specific protein.

3. **The Reviewer commented:**

***L383-384. ‘the pipeline identified 25 UTI89 proteins potentially involved in lipopolysaccharide biosynthesis/GO:0009103’. The authors should cross check these hits against previous functional genomic screens (e.g. PMID: 24098145; Phan et al 2013).***

We thank the reviewer for this helpful suggestion. We re-examined the 25 UTI89 proteins assigned exclusively by StarFunc to GO:0009103 (lipopolysaccharide biosynthetic process). Importantly, many of these do not represent entirely novel functional assignments but are encoded by genes with previously characterized roles in lipid A, KDO, heptose, O-antigen, and LPS core biosynthesis, including *lpxA, lpxK, kdsA, kdsB, hldE, hldD, lpcA, yaeD, yrbH, galU,* and *rfbC*, as well as several *rfa/waa* genes (*rfaC*, *rfaF*, *rfaG*, *rafI*, *rfaJ*, *rfaP*, *rfaQ*, *rfaY*, and *waaW*). Thus, this enrichment largely recapitulates established LPS biology rather than representing a set of exclusively novel predictions. We have therefore revised the manuscript to distinguish well-established LPS biosynthesis genes from a smaller set of additional candidates that may warrant further experimental validation. We also note that previous functional genomic studies in related UPEC strains, such as Phan *et al.* (2013), support the importance of cell-envelope and surface-polysaccharide pathways in bacterial fitness and host-associated phenotypes. In addition, the very fact that the genes identified by StarFunc as having this role often appear in prior studies, but do not have the appropriate annotations in the UniProt database, demonstrate a major aspect of the utility of our pipeline: namely, giving correct and complete annotations even in cases where current annotation databases may not yet completely represent our state of knowledge.

Using the pipeline, we identified additional UTI89 proteins associated with several biologically relevant functions, including “lipopolysaccharide biosynthesis/GO:0009103”, “symbiont-mediated perturbation of the host endomembrane system/GO:0052025”, and “mannose PTS transporter activity /GO:0022870”. For lipopolysaccharide biosynthesis, many of the assigned proteins correspond to genes with previously characterized roles in LPS biosynthesis, including *hldE*, *hldD*, *lpxA*, *lpxK*, *lpcA*, *yaeD*, *yrbH*, *kdsA*, *kdsB*, *galU*, *rfbC*, and multiple *rfa*/*waa* core biosynthesis genes (*rfaC*, *rfaF*, *rfaG*, *rafI*, *rfaJ*, *rfaP*, *rfaQ*, *rfaY*, and *waaW*), whereas the remaining predictions include additional candidates that may warrant further investigation. Consistent with the biological relevance of these pathways, previous functional genomic studies in related UPEC backgrounds have also highlighted the importance of cell-envelope and surface-polysaccharide functions in bacterial fitness and host-associated phenotypes [1]. The predictions not only supplement existing function annotations but also reveal uncharacterized proteins that may play crucial roles in UTI89’s metabolism, environmental adaptation, and virulence.

4. **The Reviewer commented:**

***L497-498. ‘provides insight into how the pathogen may adapt to oxygen-limited environments in the urinary tract… Would oxygen-limited environments in gut reservoirs be more appropriate here?***

We thank the reviewer for this helpful suggestion. We agree that oxygen-limited conditions in gut reservoirs provide a more appropriate biological context for the predicted anaerobic respiration-related proteins. We have therefore revised the sentence according to your suggestion.

The prediction that UTI89 encodes anaerobic respiration-related proteins such as *HybC* provides insight into how the pathogen may adapt to oxygen-limited conditions in gut reservoirs, potentially facilitating its persistence in this niche. Our finding also suggests potential avenues for experimental validation and therapeutic intervention. In addition, this also shows the pipeline’s utility in discovering potential functions and providing valuable candidates for further experimental validation (and, importantly, providing those annotations without manual intervention).

5. **The Reviewer commented:**

***L510-513. ‘For plasmid-encoded proteins, 99% are poorly annotated, and none received an annotation score above 4, indicating that plasmid genes remain understudied and limited annotations were provided in UniProt.’ Is this true for conjugation genes. Some comment would be informative given their importance in AMR gene transfer.***

We thank the reviewer for raising this important point. We agree that the overall low annotation scores of plasmid-encoded proteins should not be interpreted as indicating that all plasmid-borne functions are equally understudied. To clarify this issue, we separately examined the conjugation-related proteins encoded by the UTI89 virulence plasmid. Of the 33 identifiable *FinO*/*Tra*/*Trb*-associated proteins, 30 were assigned an annotation score of 1 in both the original and updated UniProt annotations, whereas only three proteins (*FinO*, *TraT*, and *TraA*) received a score of 2. Thus, the overall trend of low annotation scores also applies to conjugation-related proteins. However, these low scores do not indicate that their general biological roles are entirely unknown; rather, they reflect the sparse or nonspecific UniProt/GO annotations currently available for these proteins despite their broadly recognized roles in conjugative transfer. We have revised the manuscript accordingly to distinguish limited annotation specificity from a lack of biological knowledge or importance.

We further assessed the annotation coverage of protein-coding genes across three distinct subsets of the *E. coli* UTI89 genome on different annotation scores. These subsets include: 143 genes located on plasmid (**Figure 3C**); 4,915 chromosomal genes excluding phage-derived sequences (**Figure 3D**); 277 phage-derived genes (**Figure 3E**). The results indicate that StarFunc demonstrates a pronounced advantage in annotating poorly characterized proteins. For plasmid-encoded proteins, 99% are poorly annotated, and none received an annotation score above 4, indicating that plasmid genes remain understudied and limited annotations were provided in UniProt. Notably, this trend also applies to plasmid-borne conjugation genes: most recognizable components of the *Tra*/*Trb* transfer machinery on the UTI89 plasmid retain UniProt annotation scores of only 1 (30 genes) or 2 (3 genes), suggesting that low scores here primarily reflect limited annotation specificity rather than lack of biological importance. The low UniProt scores may also reflect a difficulty in the overall structure of annotation transfer, in which the existing methods of curation and annotation transfer may under-estimate the state of knowledge about a given protein (A clear similar example can be seen even in *E. coli* K12, where the flagellin protein FliC has only a UniProt score of 3/5, as many of the key experiments defining our state of knowledge regarding that protein have been performed in closely related organisms such as *Salmonella*, for which the FliC protein has a UniProt score of 4/5 despite the obvious homology). By contrast, StarFunc improves the functional annotation of these poorly annotated proteins, assigning at least one specific GO term in the BP, CC, and MF aspects to 47.8%, 98.5%, and 41.7% of proteins with an annotation score of 1, respectively. Across plasmid, chromosomal, and phage-derived genes, StarFunc substantially expands GO term coverage beyond what is provided by UniProt. These findings further highlight the utility of StarFunc in extending and complementing existing annotations, particularly for genes of pathogenic relevance, where its predictions may yield novel biological insights.

6. **The Reviewer commented:**

***L725-727. ‘thus, our predictions reveal a wealth of new functionalities related both to the interface of UTI89 with the hosts that it parasitizes, and at the same time, with the viruses that parasitize it.’ I found the term ‘parasitize’ misleading. UTI89 survives many host insults and the viruses are prophages. Consider rewording.***

We thank the reviewer for this comment. We agree that the term “parasitize” was misleading in this context. We have revised the text to describe these predictions more precisely as relating to UTI89-host interactions and prophage-associated functions encoded within the genome.

Taken together, this indicates that poorly annotated proteins are not functionally random; instead, they are significantly enriched for biologically coherent GO terms, particularly those associated with phage-related and host-colonization processes. Thus, our predictions reveal numerous previously unannotated functions related to UTI89-host interactions and prophage-associated processes in the UTI89 genome.

7. **The Reviewer commented:**

***The analysis of genes encoding viral proteins was interesting. Are there specific viral proteome databases that could also be examined in these analyses?***

We thank the reviewer for this helpful suggestion. We agree that dedicated viral or phage-focused protein resources could provide an additional layer of validation for the viral/prophage-related signals identified here. In the current study, we used PHASTER to identify prophage-associated genes and relied on UniProt- and StarFunc-derived annotations to assess their functional enrichment. Our main goal was to evaluate the ability of the general proteome-wide pipeline to recover biologically meaningful functions across the entire UTI89 proteome, including prophage-associated genes.
