# Supplementary material for: Whole-proteome structure/function prediction in uropathogenic E. coli reveals previously missed host-microbe and microbe-phage interaction pathways
Source: PLoS Genet. 2026 Nov 3;22(11):e1012187. doi: 10.1371/journal.pgen.1012187 (PMC13750657; doi:10.1371/journal.pgen.1012187)
Supplement: Peer review history

# Peer review: Decision Letter 0

**DOI:** 10.1371/journal.pgen.1012187.r001

**Authors:** Sean Crosson, Xavier Didelot

13 Jul 2026

PGENETICS-D-26-00565

Whole-proteome structure/function prediction in Uropathogenic E. coli

reveals previously missed host-microbe and microbe-phage interaction pathways

PLOS Genetics

Dear Dr. Freddolino,

Thank you for submitting your manuscript to PLOS Genetics. After careful consideration, we feel that it has merit but does not fully meet PLOS Genetics's publication criteria as it currently stands. Therefore, we invite you to submit a revised version of the manuscript that addresses the points raised during the review process.

Please submit your revised manuscript by Sep 11 2026 11:59PM. If you will need more time than this to complete your revisions, please reply to this message or contact the journal office at plosgenetics@plos.org. When you're ready to submit your revision, log on to https://www.editorialmanager.com/pgenetics/ and select the 'Submissions Needing Revision' folder to locate your manuscript file.

Please include the following items when submitting your revised manuscript:

* A letter that responds to each point raised by the editor and reviewer(s). You should upload this letter as a separate file labeled 'Response to Reviewers'. This file does not need to include responses to any formatting updates and technical items listed in the 'Journal Requirements' section below.

* A marked-up copy of your manuscript that highlights changes made to the original version. You should upload this as a separate file labeled 'Revised Manuscript with Track Changes'.

* An unmarked version of your revised paper without tracked changes. You should upload this as a separate file labeled 'Manuscript'.

If you would like to make changes to your financial disclosure, competing interests statement, or data availability statement, please make these updates within the submission form at the time of resubmission. Guidelines for resubmitting your figure files are available below the reviewer comments at the end of this letter.

As the corresponding author, your ORCID iD is verified in the submission system and will appear in the published article. PLOS supports the use of ORCID, and we encourage all coauthors to register for an ORCID iD and use it as well. Please encourage your coauthors to verify their ORCID iD within the submission system before final acceptance, as unverified ORCID iDs will not appear in the published article. Only the individual author can complete the verification step; PLOS staff cannot verify ORCID iDs on behalf of authors.

We look forward to receiving your revised manuscript.

Kind regards,

Xavier Didelot

Academic Editor

PLOS Genetics

Sean Crosson

Section Editor

PLOS Genetics

Aimée Dudley

Editor-in-Chief

PLOS Genetics

Anne Goriely

Editor-in-Chief

PLOS Genetics

**Additional Editor Comments (if provided):**

**Journal Requirements:**

If the reviewer comments include a recommendation to cite specific previously published works, please review and evaluate these publications to determine whether they are relevant and should be cited. There is no requirement to cite these works unless the editor has indicated otherwise.

1) Please ensure that the CRediT author contributions listed for every co-author are completed accurately and in full.

At this stage, the following Authors/Authors require contributions: Chunxiang Peng, Henry Schreiber, Chengxin Zhang, Quancheng Liu, Scott Hultgren, and Lydia Freddolino. Please ensure that the full contributions of each author are acknowledged in the "Add/Edit/Remove Authors" section of our submission form.

The list of CRediT author contributions may be found here: https://journals.plos.org/plosgenetics/s/authorship#loc-author-contributions

2) We ask that a manuscript source file is provided at Revision. Please upload your manuscript file as a .doc, .docx, .rtf or .tex. If you are providing a .tex file, please upload it under the item type u2018LaTeX Source Fileu2019 and leave your .pdf version as the item type u2018Manuscriptu2019.

3) Please provide an Author Summary. This should appear in your manuscript between the Abstract (if applicable) and the Introduction, and should be 150-200 words long. The aim should be to make your findings accessible to a wide audience that includes both scientists and non-scientists. Sample summaries can be found on our website under Submission Guidelines:

https://journals.plos.org/plosgenetics/s/submission-guidelines#loc-parts-of-a-submission

4) Please upload all main figures as separate Figure files in .tif or .eps format. For more information about how to convert and format your figure files please see our guidelines:

https://journals.plos.org/plosgenetics/s/figures

5) Please upload a copy of Figures FIG 1, 2A-F, 3A-E, 4A-C, 5A-E, 6A-D, and 7A-D which you refer to in your text on pages 12, 16, 21, 23, 26, 33, and 35. Or, if the figure is no longer to be included as part of the submission please remove all reference to it within the text.

6) We have noticed that you have uploaded Supporting Information files, but you have not included a list of legends. Please add a full list of legends for your Supporting Information files after the references list.

7) Please amend your detailed Financial Disclosure statement. This is published with the article. It must therefore be completed in full sentences and contain the exact wording you wish to be published.

i) State the initials, alongside each funding source, of each author to receive each grant. For example: "This work was supported by the National Institutes of Health (####### to AM; ###### to CJ) and the National Science Foundation (###### to AM)."

ii) State what role the funders took in the study. If the funders had no role in your study, please state: "The funders had no role in study design, data collection and analysis, decision to publish, or preparation of the manuscript."

iii) If any authors received a salary from any of your funders, please state which authors and which funders..

If you did not receive any funding for this study, please simply state: u201cThe authors received no specific funding for this work.u201d

8)We have amended your Competing Interest statement to comply with journal style. We kindly ask that you double check the statement and let us know if anything is incorrect

**Reviewers' comments:**

Reviewer's Responses to Questions

**Comments to the Authors:**

**Please note here if the review is uploaded as an attachment.**

Reviewer #1: General comments:

The manuscript in general is well written, and would be of interest to the scientific community, especially those working with poorly annotated or non-model organisms. The level of functional validation is limited but consistent with publications of a similar nature. The experiments for functional validation are also well designed. I have some concerns regarding the accuracy of the model and the lack of comparison to other state of the art methods.

Specific comments:

1. Figure 2 B, StarFunc alone predicts between ~23 and 44 % of all Go-Terms. It would be useful for readers to assess the reliability of StarFunc predictions at scale if the authors showed a Cscore distribution for the GO-Terms predicted by StarFunc alone, and those present in the UniProt annotation and predicted by StarFunc (both).

2. Similarly, Figure 2 B shows that StarFunc fails to predict UniProt Go annotations in between ~ 25 and 33% of cases. In cases where StarFunc failed to predict the correct UniProt annotation (UniProt), it would be useful to compare the Cscore of the UniProt annotations as predicted by StarFunc for a given protein to that of the reported annotations. Essentially a rank difference between the uniport annotation vs StarFunc annotation.

3. In Figure 3, the authors compare their StarFunc pipeline to two alternatives. The authors also claim to meet or exceed state of the art methods with StarFunc, however the authors fail to compare to more recent pipelines, such as NetGO 4.0 or GOAnnotator.

4. In Figure 3, given the relatively modest improvements in wFmax observed for StarFunc, it would help the reader to also compare the computational load between methods tested. A metric such as sequences annotation per time, may be a useful metric for users who may have large sequence databases to predict.

5. In line 382, the authors mistakenly refer to Figure 1, where it should be Figure 2

6. The Authors should provide the date of access to the UniProt data used in this study.

Reviewer #2: This manuscript describes the development and pilot application of an automated structure-based protein functional annotation pipeline for bacteria. The authors then applied this pipeline to predict protein structure and function characteristics across the entire proteome of the UPEC reference strain UTI89. Finally, the authors used their findings to establish a UTI89 structure-function database as an online resource to enable systematic function annotations based on proteome-wide structure modelling for this strain.

Overall, the data presented are sound, the manuscript is very well written, and work will be of enormous interest to other researchers in the field.

Comments

The study is well designed and methodologically sound. I have no substantive concerns regarding the data, and the major conclusions are appropriately supported by the evidence presented.

L80. Do you mean the ‘structure and function of bacterial ‘proteins’ essential for…

L330. ‘only 4 proteins were assigned an annotation score of 5’. I found this hard to understand given we know so much about E. coli proteins. Further explanation would be helpful.

L383-384. ‘the pipeline identified 25 UTI89 proteins potentially involved in lipopolysaccharide biosynthesis/GO:0009103’. The authors should cross check these hits against previous functional genomic screens (e.g. PMID: 24098145; Phan et al 2013).

L497-498. ‘provides insight into how the pathogen may adapt to oxygen-limited environments in the urinary tract… Would oxygen-limited environments in gut reservoirs be more appropriate here?

L510-513. ‘For plasmid-encoded proteins, 99% are poorly annotated, and none received an annotation score above 4, indicating that plasmid genes remain understudied and limited annotations were provided in UniProt.’ Is this true for conjugation genes. Some comment would be informative given their importance in AMR gene transfer.

L725-727. ‘thus, our predictions reveal a wealth of new functionalities related both to the interface of UTI89 with the hosts that it parasitizes, and at the same time, with the viruses that parasitize it.’ I found the term ‘parasitize’ misleading. UTI89 survives many host insults and the viruses are prophages. Consider rewording.

The analysis of genes encoding viral proteins was interesting. Are there specific viral proteome databases that could also be examined in these analyses?

**********

**Have all data underlying the figures and results presented in the manuscript been provided?**

Large-scale datasets should be made available via a public repository as described in the *PLOS Genetics*  data availability policy, and numerical data that underlies graphs or summary statistics should be provided in spreadsheet form as supporting information.

Reviewer #1: Yes

Reviewer #2: Yes

**********

PLOS authors have the option to publish the peer review history of their article (what does this mean?). If published, this will include your full peer review and any attached files.

If you choose “no”, your identity will remain anonymous but your review may still be made public.

**Do you want your identity to be public for this peer review?**  For information about this choice, including consent withdrawal, please see our Privacy Policy.

Reviewer #1: No

Reviewer #2: **Yes:**  Mark Schembri

[NOTE: If reviewer comments were submitted as an attachment file, they will be attached to this email and accessible via the submission site. Please log into your account, locate the manuscript record, and check for the action link "View Attachments". If this link does not appear, there are no attachment files.]

**Figure resubmission:**

**Reproducibility:**

To enhance the reproducibility of your results, we recommend that authors of applicable studies deposit laboratory protocols in protocols.io, where a protocol can be assigned its own identifier (DOI) such that it can be cited independently in the future. Additionally, PLOS ONE offers an option to publish peer-reviewed clinical study protocols. Read more information on sharing protocols at https://plos.org/protocols?utm_medium=editorial-email&utm_source=authorletters&utm_campaign=protocols

# Author comment: Author response to Decision Letter 1

**DOI:** 10.1371/journal.pgen.1012187.r002

11 Oct 2026

Attachment Submitted filename: ResponseToReviwers_latest_lfmod_20260922.docx

# Editor report: Decision Letter 1

**DOI:** 10.1371/journal.pgen.1012187.r003

**Authors:** Sean Crosson, Xavier Didelot

14 Oct 2026

Dear Dr Freddolino,

We are pleased to inform you that your manuscript entitled "Whole-proteome structure/function prediction in Uropathogenic E. coli reveals previously missed host-microbe and microbe-phage interaction pathways" has been editorially accepted for publication in PLOS Genetics. Congratulations!

Before your submission can be formally accepted and sent to production you will need to complete our formatting changes, which you will receive in a follow up email. Please be aware that it may take several days for you to receive this email; during this time no action is required by you. Please note: the accept date on your published article will reflect the date of this provisional acceptance, but your manuscript will not be scheduled for publication until the required changes have been made.

Once your paper is formally accepted, an uncorrected proof of your manuscript will be published online ahead of the final version, unless you’ve already opted out via the online submission form. If, for any reason, you do not want an earlier version of your manuscript published online or are unsure if you have already indicated as such, please let the journal staff know immediately at plosgenetics@plos.org.

In the meantime, please log into Editorial Manager at https://www.editorialmanager.com/pgenetics/, click the "Update My Information" link at the top of the page, and update your user information to ensure an efficient production and billing process. Note that PLOS requires an ORCID iD for all corresponding authors. Therefore, please ensure that you have an ORCID iD and that it is validated in Editorial Manager. To do this, go to ‘Update my Information’ (in the upper left-hand corner of the main menu), and click on the Fetch/Validate link next to the ORCID field.  This will take you to the ORCID site and allow you to create a new iD or authenticate a pre-existing iD in Editorial Manager.

If you have a press-related query, or would like to know about making your underlying data available (as you will be aware, this is required for publication), please see the end of this email. If your institution or institutions have a press office, please notify them about your upcoming article at this point, to enable them to help maximise its impact. Inform journal staff as soon as possible if you are preparing a press release for your article and need a publication date.

Thank you again for supporting open-access publishing; we are looking forward to publishing your work in PLOS Genetics!

Yours sincerely,

Xavier Didelot

Academic Editor

PLOS Genetics

Sean Crosson

Section Editor

PLOS Genetics

Aimée Dudley

Editor-in-Chief

PLOS Genetics

Anne Goriely

Editor-in-Chief

PLOS Genetics

www.plosgenetics.org

BlueSky: @plos.bsky.social

**Data Deposition**

If you have submitted a Research Article or Front Matter that has associated data that are not suitable for deposition in a subject-specific public repository (such as GenBank or ArrayExpress), one way to make that data available is to deposit it in the Dryad Digital Repository. As you may recall, we ask all authors to agree to make data available; this is one way to achieve that. A full list of recommended repositories can be found on our website.

The following link will take you to the Dryad record for your article, so you won't have to re‐enter its bibliographic information, and can upload your files directly:

http://datadryad.org/submit?journalID=pgenetics&manu=PGENETICS-D-26-00565R1

More information about depositing data in Dryad is available at http://www.datadryad.org/depositing. If you experience any difficulties in submitting your data, please contact help@datadryad.org for support.

Additionally, please be aware that our data availability policy requires that all numerical data underlying display items are included with the submission, and you will need to provide this before we can formally accept your manuscript, if not already present.

----------------------------------------------------

**Press Queries**

If you or your institution will be preparing press materials for this manuscript, or if you need to know your paper's publication date for media purposes, please inform the journal staff as soon as possible so that your submission can be scheduled accordingly. Your manuscript will remain under a strict press embargo until the publication date and time. This means an early version of your manuscript will not be published ahead of your final version. PLOS Genetics may also choose to issue a press release for your article. If there's anything the journal should know or you'd like more information, please get in touch via plosgenetics@plos.org.

# Editor report: Acceptance letter

**DOI:** 10.1371/journal.pgen.1012187.r004

**Authors:** Sean Crosson, Xavier Didelot

PGENETICS-D-26-00565R1

Whole-proteome structure/function prediction in Uropathogenic E. coli

reveals previously missed host-microbe and microbe-phage interaction pathways

Dear Dr Freddolino,

We are pleased to inform you that your manuscript entitled "Whole-proteome structure/function prediction in Uropathogenic E. coli

reveals previously missed host-microbe and microbe-phage interaction pathways" has been formally accepted for publication in PLOS Genetics! Your manuscript is now with our production department and you will be notified of the publication date in due course.

The corresponding author will soon be receiving a typeset proof for review, to ensure errors have not been introduced during production. Please review the PDF proof of your manuscript carefully, as this is the last chance to correct any errors. Please note that major changes, or those which affect the scientific understanding of the work, will likely cause delays to the publication date of your manuscript.

Soon after your final files are uploaded, unless you have opted out or your manuscript is a front-matter piece, the early version of your manuscript will be published online. The date of the early version will be your article's publication date. The final article will be published to the same URL, and all versions of the paper will be accessible to readers.

For Research Articles, you will receive an invoice from PLOS for your publication fee after your manuscript has reached the completed accept phase. If you receive an email requesting payment before acceptance or for any other service, this may be a phishing scheme. Learn how to identify phishing emails and protect your accounts at https://explore.plos.org/phishing.

Thank you again for supporting PLOS Genetics and open-access publishing. We are looking forward to publishing your work!

With kind regards,

Anitha Samidurai

PLOS Genetics

On behalf of:

The PLOS Genetics Team

Carlyle House, Carlyle Road, Cambridge CB4 3DN | United Kingdom

plosgenetics@plos.org | +44 (0) 1223-442823

plosgenetics.org | Twitter: @PLOSGenetics
